# Rising Incidence and Spatiotemporal Dynamics of Emerging and Reemerging Arboviruses in Brazil

**DOI:** 10.3390/v17020158

**Published:** 2025-01-24

**Authors:** Matheus Daudt-Lemos, Alice Ramos-Silva, Renan Faustino, Tatiana Guimarães de Noronha, Renata Artimos de Oliveira Vianna, Mauro Jorge Cabral-Castro, Claudete Aparecida Araújo Cardoso, Andrea Alice Silva, Fabiana Rabe Carvalho

**Affiliations:** 1 Multiuser Laboratory for Research Support in Nephrology and Medical Sciences, Faculty of Medicine, Universidade Federal Fluminense, Niterói 24033900, RJ, Brazil; mdaudt@id.uff.br (M.D.-L.); aliceramos@id.uff.br (A.R.-S.); renan.faustino@ioc.fiocruz.br (R.F.); maurojorge@id.uff.br (M.J.C.-C.); claudetecardoso@id.uff.br (C.A.A.C.); 2 Laboratory of Respiratory Viruses, Exanthematics, Enteroviruses and Viral Emergencies, Instituto Oswaldo Cruz, Fundação Oswaldo Cruz, Rio de Janeiro 21040900, RJ, Brazil; 3 Faculty of Medicine, Universidade Federal Fluminense, Niterói 24033900, RJ, Brazil or tatiana.denoronha@paediatrics.ox.ac.uk (T.G.d.N.); renata_artimos@id.uff.br (R.A.d.O.V.); 4 Department of Paediatrics, University of Oxford, Oxford OX3 7LE, UK; 5 Graduate Program in Pathology, Faculty of Medicine, Universidade Federal Fluminense, Niterói 24033900, RJ, Brazil

**Keywords:** emergent pathogen, Zika virus, chikungunya virus, dengue, Oropouche virus

## Abstract

Background: Brazil has witnessed the co-circulation of dengue virus (DENV), Zika virus (ZIKV), and chikungunya virus (CHIKV), with outbreaks exacerbated by environmental factors, social determinants, and poor sanitation. The recent re-emergence of Oropouche virus (OROV) has added complexity to vector control strategies, emphasizing the need for integrated approaches to curb arboviruses spread. We aimed to analyze temporal trends and spatial distributions with national scope of these emerging arboviruses. Methods: An ecological study using data from the Brazilian Notifiable Diseases Information System the period from 2023 to 2024 was undertaken. Temporal trends were evaluated using Joinpoint regression, while spatial analysis was conducted using Moran’s I, and local indicators of spatial association. Results: Dengue fever cases increased by 322%, while Oropouche fever (OF) increased by 300%. The states of Amazonas and Espírito Santo reported increases in OF cases. Moran’s I test revealed spatial clustering of DENV and CHIKV. Two municipalities in the state of Mato Grosso do Sul showed cocirculation of DENV, CHIKV, and ZIKV. Conclusions: This study identified a surge in arbovirus cases between 2023 and 2024, with peak incidences from January to March and October to December, linked to favorable climatic conditions. Clustering patterns and co-circulation of arboviruses highlight the need for tailored control and prevention strategies and targeted interventions to mitigate their impact.

## 1. Introduction

Arboviral diseases, including dengue virus (DENV), Zika virus (ZIKV), and chikungunya virus (CHIKV), represent significant public health challenges in tropical and subtropical regions of Brazil. These viruses share common transmission vectors, *Aedes aegypti* and *Aedes albopictus*, which are predominantly distributed in urban areas [1]. The reliance on these vectors complicates accurate clinical diagnosis due to overlapping symptoms such as fever, rash, and arthralgia, while their wide distribution facilitates rapid dissemination and amplifies public health impacts [2]. Despite these shared characteristics, arboviruses exhibit unique epidemiological profiles influenced by distinct ecological and environmental factors, such as temperature, precipitation, and altitude, which contribute to variations in their transmission dynamics [3]. Furthermore, simultaneous exposure to multiple arboviruses facilitates simultaneous transmission, thereby increasing the epidemiological complexity of these infections [4]

Historically, regions such as northeast Brazil and the Amazon have experienced the co-circulation of multiple arboviruses [5]. In 2024, the emergence of Oropouche fever marked a critical addition to the arboviral landscape. Oropouche fever is caused by an arbovirus transmitted by *Culicoides paraensis*, a biting midge with distinct ecological and behavioral characteristics [6]. Unlike Aedes species, the control of Culicoides populations is particularly challenging due to their breeding in decaying organic matter and their smaller, less conspicuous size, which complicates surveillance [7,8]. Their biting activity predominantly occurs at dusk and nighttime, further hindering the timing of effective control measures [9,10]. Between January and October 2024, over 8000 cases of Oropouche fever were reported, marking its rapid spread beyond the Amazon region. This geographic expansion underscores the need for specialized prevention strategies, robust containment measures, and public awareness campaigns tailored to the unique characteristics of Culicoides vectors [11]. Recent reports of vertical transmission in Pernambuco have raised concerns, including the first confirmed fetal death due to Oropouche fever and cases of congenital anomalies such as microcephaly [12,13].

In addition to DENV, ZIKV, CHIKV, and Oropouche virus, other arboviruses, such as yellow fever virus (YFV) and Mayaro virus (MAYV), contribute to the complexity of arbovirus surveillance and control in Brazil. These viruses are transmitted by mosquitoes of the genera *Haemagogus* and *Sabethes* and involve both wild and urban transmission cycles, requiring multifaceted strategies for effective management [12,14]. Innovative approaches, such as the World Mosquito Program (WMP) and the use of *Wolbachia*-infected mosquitoes, aim to provide sustainable solutions for reducing arbovirus transmission. However, vaccines remain unavailable for ZIKV, CHIKV, Oropouche, and MAYV, highlighting the need for alternative prevention strategies. Brazil’s Unified Health System (SUS) currently offers free vaccines for YFV and a tetravalent dengue vaccine for children aged 10 to 14 in high-incidence regions, though these face logistical and immunological challenges [10,11].

The co-circulation of these arboviruses, along with the rapid spread of Oropouche fever, underscores the critical importance of robust arbovirus surveillance [5,6]. Effective monitoring allows timely outbreak detection, guides vector control strategies, and strengthens healthcare readiness to mitigate disease impacts [7,15]. Understanding the spatial distribution and ecological drivers of arbovirus transmission is essential for developing targeted preventive measures and raising public awareness, particularly among vulnerable populations [16]. In this context, the present study aimed to evaluate the spatial distribution of arboviruses in Brazil and identify periods of increased incidence. Our findings contribute to public health by informing future vector control strategies and enhancing clinical management of arboviral diseases [17].

## 2. Materials and Methods

### 2.1. Study Design

We conducted a population-based ecological study using data extracted from the Notifiable Diseases Information System (SINAN) database (Minister of Health, Brazil), covering the period from January 2023 to September 2024. The study period was specifically chosen to exceed one year, as 2023 marked the emergence of Oropouche cases outside the Amazon region. This extended timeframe enabled a comprehensive analysis of the virus dynamics, including its geographic spread, potential epidemic patterns, and seasonal variations. The study focused on case records of Oropouche, Zika, chikungunya, yellow fever, and dengue.

### 2.2. Data Sources

The data analyzed in this study were obtained from the SINAN database, accessible through the DATASUS platform (Brazil). Files were downloaded in .dbc format for the study period and processed using RStudio. Each entry represented a reported case of arboviral diseases, including dengue, Zika, chikungunya, and yellow fever. The data were aggregated at the municipal level and adjusted for population size to enable per capita analysis.

For Oropouche virus cases, which are not yet included in the .dbc files of SINAN, data were manually collected from the surveillance dashboard provided by ANVISA (National Health Surveillance Agency, Brazil). Consistency checks were conducted to identify discrepancies, such as mismatches between reported ages and dates of birth. Duplicate entries were also removed to ensure the accuracy and reliability of the dataset.

### 2.3. Statistical Analysis

The incidence was calculated by dividing the number of new cases reported during the study period by the population of interest, adjusted per 100,000 inhabitants. For Zika, chikungunya, and dengue, we performed a comprehensive spatial and temporal analysis using RStudio and the spatial package to explore the geographic distribution of cases and temporal trends. Spatial autocorrelation was evaluated using Moran’s I test, a statistical method that quantifies the degree of spatial clustering of a variable. Moran’s I values range from −1 to +1, where positive values indicate spatial clustering (similar values are closer together), negative values suggest dispersion (dissimilar values are closer together), and values near zero imply a random spatial distribution. This test is commonly used to assess whether disease cases are distributed randomly or exhibit geographic patterns that may require targeted interventions. Additionally, local indicators of spatial association (LISA) were applied to identify and map areas with significantly high or low incidence rates, highlighting geographic hotspots and regions potentially requiring targeted public health interventions.

To analyze the temporal trends, we employed the Joinpoint Regression Program, a specialized tool for detecting significant changes in time-series data. This method identifies points where the direction or magnitude of trends shift significantly. Temporal trends were quantified using the weekly percent change (WPC) and the average annual percent change (AAPC). The WPC reflects the relative change in case incidence between consecutive epidemiological weeks, providing a granular view of short-term trends. The AAPC, in contrast, aggregates these weekly variations to estimate the overall percentage change per year, offering a broader perspective on long-term trends.

### 2.4. Ethical Considerations

This study utilized anonymized, publicly accessible data from the SINAN database, ensuring full compliance with ethical guidelines for secondary data usage. We utilized publicly available data that does not include patient identifiers. In accordance with Brazilian law, this type of research does not require approval from an ethics and research committee.

## 3. Results

### 3.1. Overview of Arbovirus Incidence

Brazil has a territory of 8.5 million square kilometers and approximately 203 million inhabitants. The country is organized into 5 macro-regions, divided into 27 federative units, and subdivided into 5570 municipalities.

The analysis revealed a significant increase in arbovirus cases in Brazil between 2023 and 2024. The total number of cases rose from 1,786,297 in 2023 to 6,790,276 in 2024, representing a global increase of over 280%. Among the arboviruses studied, dengue showed the highest percentage increase at 322%, followed by Oropouche with a 300% increase, Chikungunya with 49.9%, and Zika with 43.1%. Despite the availability of vaccines, yellow fever remains a concern, with six cases reported in 2023. All cases involved young male adults, and the disease exhibited a high mortality rate of 66.7%.

### 3.2. Geographical Distribution

#### 3.2.1. Regional Trends

The surge in arbovirus cases was most notable in the North region, where the state of Amazonas led in absolute case numbers. Significant increases in Oropouche infections were observed in Amapá (AP) and Espírito Santo (*p* < 0.001). Tocantins (TO) reported 1874 new Zika cases, despite having no cases in 2023. In contrast, some regions reported no cases of certain arboviruses in 2024, such as the Federal District for dengue and Mato Grosso do Sul for chikungunya.

#### 3.2.2. Spatial Analysis

**Figure 1:**
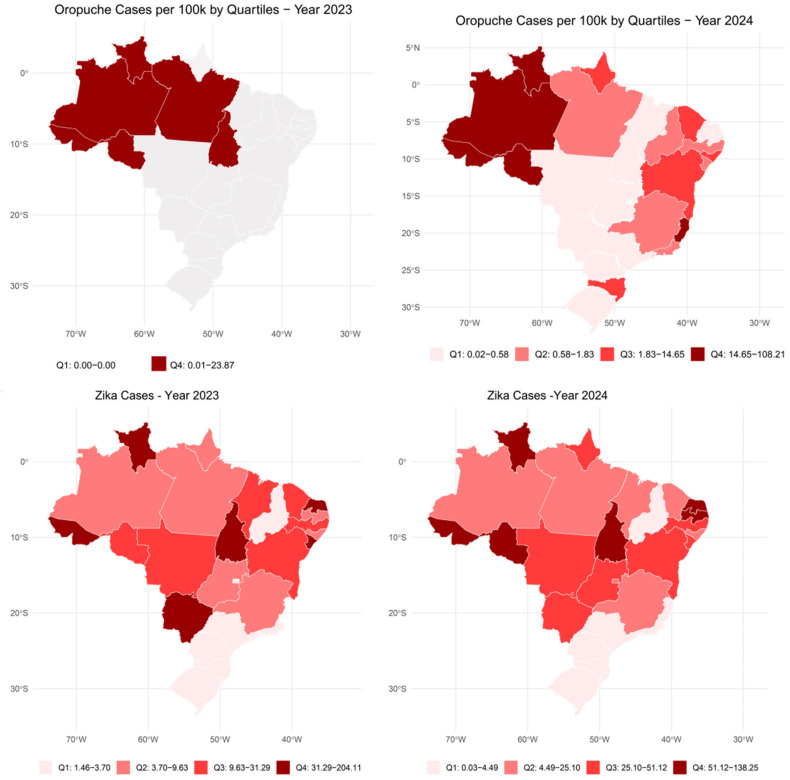
Distribution of Oropouche and Zika cases per 100,000 inhabitants from January 2023 to September 2024.

**Figure 2:**
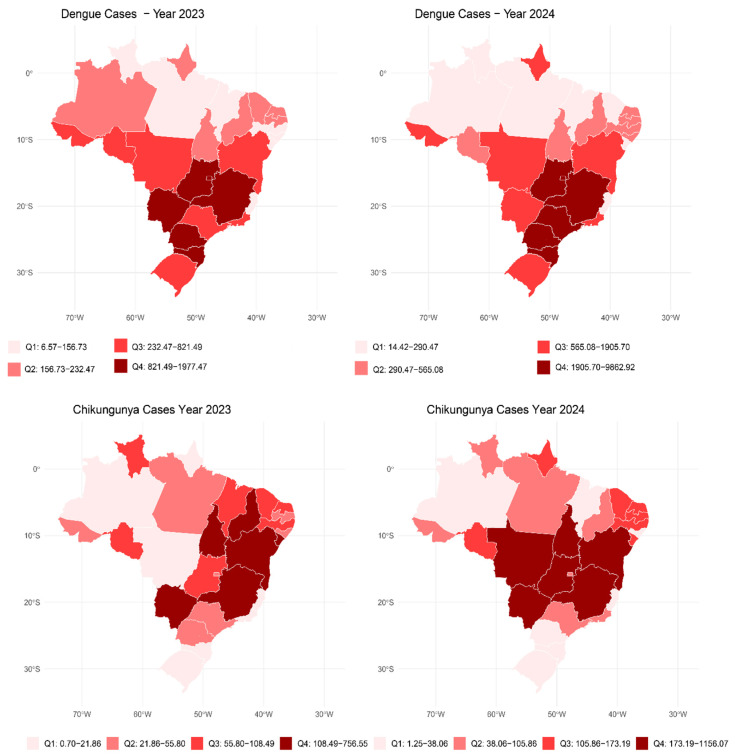
Distribution of dengue and chikungunya cases per 100,000 inhabitants from January 2023 to September 2024.

Choropleth maps (Figure 1 and Figure 2) illustrate arbovirus case distributions, with Oropouche cases concentrated in the north region, while dengue incidence was higher in the southeast and south regions. Spatial autocorrelation analysis using Moran’s I revealed significant clustering patterns for multiple arboviruses. Dengue exhibited the strongest clustering (Moran’s I = 0.49, *p* < 0.001), followed by chikungunya (Moran’s I = 0.38, *p* < 0.0001) and Zika (Moran’s I = 0.086, *p* = 0.003). These findings suggest that regional factors, such as urban infrastructure, vector control strategies, and environmental conditions, play pivotal roles in shaping disease transmission.

### 3.3. Geographic Clusters and Co-Circulation

**Figure 3:**
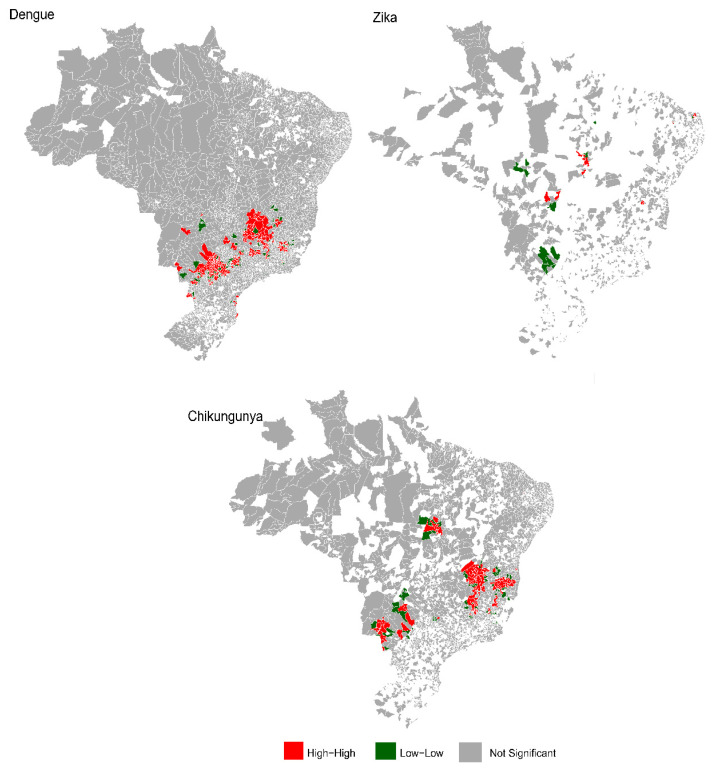
Spatial distribution of hotspots for dengue, Zika, and chikungunya in Brazil. Red (high–high): Municipalities with high incidence located in regions of high incidence for each arbovirus; Green (low–low): Municipalities with low incidence situated in areas of low incidence for each arbovirus; Gray (not significant): Municipalities with non-significant spatial patterns.

Clusters of arbovirus co-circulation were identified in various regions. In Mato Grosso do Sul, municipalities such as Batayporã and Três Lagoas reported simultaneous circulation of dengue, chikungunya, and Zika. Co-circulation of Zika and chikungunya was observed in municipalities in Tocantins, Bahia, and Mato Grosso do Sul, while clusters involving dengue and chikungunya were detected in 25 municipalities in Minas Gerais, as well as in São Paulo, Mato Grosso do Sul, and Goiás. Low–low dengue clusters surrounded by high–high clusters further highlighted complex spatial patterns in disease transmission. Figure 3 provides a detailed representation of these patterns.

### 3.4. Temporal Patterns

Temporal analyses were conducted using population-weighted national data, revealing significant increases in arbovirus incidence during specific periods. Weekly percentage change (WPC) analyses indicated national increases for dengue (WPC = 26.47, *p* < 0.0001), Zika (WPC = 15.60, *p* < 0.000001), chikungunya (WPC = 11.14, *p* < 0.000001), and Oropouche (WPC = 34.20, *p* < 0.0008), particularly from January to March 2023 and October 2023 to March 2024.

**Figure 4:**
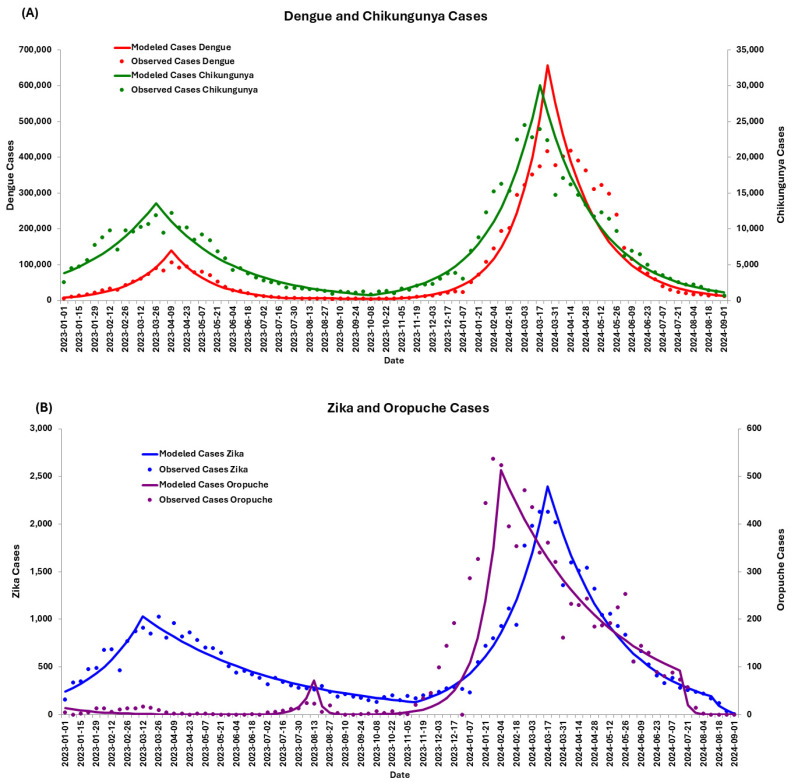
Temporal trends of arbovirus cases in Brazil: Observed and modeled data by Joinpoint regression. Panel (**A**) shows dengue and Chikungunya cases, with red dots and lines representing observed and modeled cases for dengue, and green dots and lines representing observed and modeled cases for Chikungunya. Panel (**B**) depicts Zika and Oropouche cases, with blue dots and lines representing observed and modeled cases for Zika, and purple dots and lines representing observed and modeled cases for Oropouche. The *X*-axis represents epidemiological weeks from January 2023 to September 2024, while the *Y*-axis shows case counts, using primary and secondary axes for each arbovirus.

Between January 2023 and September 2024, the average annual percentage change (AAPC) varied among arboviruses. Dengue exhibited a slight but statistically significant increase (AAPC = 0.53, *p* < 0.000001), while Zika (AAPC = −2.0012, *p* < 0.000001) and chikungunya (AAPC = −1.4139, *p* < 0.000001) showed significant decreases. Oropouche fever demonstrated a variable trend, with an AAPC of −5.5083 (*p* < 0.0001), reflecting inconsistent patterns over time. Figure 4 illustrates observed and modeled case data for these arboviruses across epidemiological weeks. The WPC of arbovirus infection in Brazil between 2023 and 2024 is detailed in Table S1 of the Supplemental Material.

### 3.5. Integrated Analysis of Geographic and Temporal Patterns

The application of spatial autocorrelation (Moran I) and temporal trend analyses (WPC, AAPC) provided insights into the geographic and temporal dynamics of arboviruses in Brazil. Spatial analysis identified regional clusters influenced by environmental and socioeconomic factors, while temporal analysis highlighted periods of heightened arbovirus incidence. For example, co-circulation hotspots were identified in municipalities like Batayporã and Três Lagoas, indicating areas requiring intensified vector control efforts. The integration of these methodologies facilitated the identification of patterns and trends, underscoring their value in designing targeted public health interventions and optimizing resource allocation

### 3.6. Implications for Public Health

This comprehensive analysis highlights the importance of integrating geographic and temporal methodologies to improve epidemiological surveillance and response strategies. Regional differences in infrastructure, vector control, and environmental factors must be addressed to combat arbovirus outbreaks effectively. These findings provide evidence to guide resource allocation and design targeted interventions, emphasizing the need for sustained efforts to mitigate the impact of arboviruses in Brazil.

## 4. Discussion

The findings of this study underscore the substantial increase in the incidence of arboviral diseases in Brazil between 2023 and 2024, with a global surge of over 280% in reported cases. Dengue exhibited the highest percentage increase at 322%, reflecting ongoing vulnerability to the disease despite the availability of vaccines. The sharp rise in Oropouche cases (300%) is also a critical warning, considering its geographic spread beyond the Amazon region and the identification of new epidemic clusters. Although the increase in dengue cases occurred during a previously known epidemic, our method adds value by exploring integrated geographic and temporal patterns, as well as identifying co-circulation clusters [18,19]. The identification of clusters demonstrates how spatial autocorrelation can pinpoint areas requiring intensified interventions. These findings align with the observed increases in temporal trends during the January to March 2023 period, reflecting the influence of seasonal factors on arbovirus transmission. These analyses not only confirm the rise in incidence but also contribute to investigating the factors driving transmission in different regions, thereby assisting in the planning of municipal-level intervention and vector control strategies. Furthermore, temporal analyses support the prediction of potential future epidemics based on retrospective data, enabling the allocation of resources and efforts to critical locations and time periods [19].

Recent studies corroborate our findings, emphasizing the interplay between environmental, socioeconomic, and biological factors driving arbovirus transmission. For instance, Gräf et al. (2022) highlighted the expansion of Oropouche fever beyond its Amazonian origins [20], now detected in nine additional states. The role of seasonal rainfall in boosting mosquito breeding sites and increasing transmission risks has been well-documented, aligning with our temporal analyses [21]. Spatial clustering of dengue and chikungunya, as observed in municipalities like Batayporã and Três Lagoas, underscores the critical influence of urban infrastructure and vector ecology in shaping arbovirus dynamics [22,23]. These clusters highlight the need for integrated spatial–temporal strategies, such as those proposed by Churakov et al. (2019), to address hotspots and inform targeted public health actions [24].

We observed an increase in dengue and Oropouche infections throughout the national territory, demonstrating the magnitude and rapid spread of the disease, in addition to the easy adaptation of the virus to the climatic characteristics of the Brazilian states. Despite these developments, the consequences of coinfection and reinfection among circulating arboviruses in Brazil remain poorly understood. Due to the overlapping clinical symptoms and the short diagnostic window (1–8 days) for molecular detection of arboviruses [13], outbreaks may go unnoticed in certain regions. Given these challenges, our objective was to identify periods of heightened arbovirus incidence, aiming to support future preventive guidelines and vector control strategies. These efforts are critical as arboviruses remain a significant public health challenge in Brazil.

The mosquito, *Aedes aegypti*, is the main vector of arboviruses circulating in Brazil, such as Dengue, Chikungunya, and Zika. Studies showed that the geographic heterogeneity of arboviruses may be related to differences in the competence of mosquito populations, conceptualized by the Ross–Macdonald mathematical model [25,26]. In our study, we observed that southern states, such as Rio Grande do Sul (RS) and Paraná (PR), have few cases of chikungunya and Zika. In August 2024, the state of Paraná began releasing the first Wolbitos in the west and north regions. In other countries such as Australia, Colombia, and Indonesia, which participate in the same action to control seasonal outbreaks of arbovirus infection using Wolbitos, they observed a reduction of 65–95% in the predicted incidence of dengue [27,28,29]. In relation to the state of Santa Catarina (SC), there was an outbreak of Oropouche in 2024, with a significant increase in reported cases.

In recent years, climate change caused by human activities has directly impacted the egg hatching period and altered the distribution area of arboviruses [30]. Associated with these factors, the distribution of cases is directly related to the bionomics of infected mosquitoes, in addition to characteristics such as the estimated flight distance capacity (~3 km/day) and the total life cycle (~45 days), which are important factors for the propagation and formation of well-defined clusters [31]. In our study, we observed the formation of municipal arbovirus clusters in some municipalities, with greater emphasis on the municipalities of Batayporã and Três Lagoas (MS) that reported the simultaneous circulation of dengue, chikungunya and Zika virus. The high rainfall levels presented by these municipalities (average of 1340 to 1700 mm) are like those in the north region (1500 to 2500 mm), where the rainfall level is the highest in Brazil, which may be linked to the high circulation of these arboviruses, after the dry season [32].

No vaccines are available against the chikungunya, Oropouche, or Zika viruses. As a strategy to control arboviruses in the country, the Brazilian Ministry of Health recently pioneered (2023) the incorporation of the dengue vaccine into the Unified Health System (SUS). However, the vaccine is not universally free; it is provided at no cost only to certain priority groups, such as children and adolescents aged 10 to 14 in endemic regions, the population with the highest number of hospitalizations [33,34]. To date, more than two million children and adolescents have been immunized. In addition, recent data show the efficacy of the Phase 3 clinical trial of the chikungunya vaccine in Brazil, developed by the Butantan Institute, regardless of previous exposure to the virus [35]. In 2023, this vaccine was approved by the Food and Drug Administration (FDA) for use in the United States, being the first vaccine authorized against chikungunya, being the first vaccine authorized for use in the world against chikungunya [36].

Our study has certain limitations. Primarily, it relies on data from SINAN, Brazil’s main epidemiological surveillance system, which depends on case reporting. Despite the mandatory requirement for arbovirus notification, the high workload and extensive reporting forms may discourage compliance, leading to potential underestimation of cases [37]. Additionally, biases may arise from underreporting or inconsistent reporting practices across states. Some federative units might transmit less information due to surveillance system limitations, resource constraints, or local policies. This could create analytical artifacts, such as falsely suggesting the absence or reduced prevalence of certain viruses in these areas. To address these issues, we conducted a consistency check during data preprocessing to identify and correct potential discrepancies. Nevertheless, variations in reporting completeness remain an inherent limitation of using secondary surveillance data. Therefore, our findings should be interpreted with caution, particularly for states with historically lower data transmission rates.

Furthermore, since most cases of the disease are self-limited, the distance between homes and healthcare centers may mean that only cases that require specialized medical care are reported. This would make the scale of the problem of arbovirus spread even more critical. Another limitation of this study is the lack of public access to data on Mayaro infection. As recently published, this virus can be transmitted by the same vector as dengue, Zika, and chikungunya [38], and it would be interesting to demonstrate the potential for dissemination and urbanization of Mayaro in the last year, by carrying out a spatial analysis. Regarding Oropouche, spatial analysis was not possible due to the unavailability of data at the municipal level. Spatial regression is a statistical technique that requires the creation of a neighborhood matrix. Data aggregated at the state level such as Oropouche made it impossible to create this matrix.

## 5. Conclusions

This study analyzed the spatial distribution of arboviruses in Brazil and identified periods of heightened incidence, with a notable surge in cases between 2023 and 2024. Dengue and Oropouche exhibited the highest growth rates, characterized by heterogeneous distribution patterns likely influenced by regional and environmental factors. Temporal analysis revealed critical periods of elevated incidence, particularly from January to March and October to December, corresponding to climatic conditions conducive to vector proliferation.

The identification of clustering patterns and the co-circulation of multiple arboviruses, as observed in municipalities within Mato Grosso do Sul, underscores the urgent need for tailored control and prevention strategies. Moreover, this study emphasizes the importance of robust surveillance systems and innovative approaches to mitigate the impacts of arboviral outbreaks.

Finally, these findings provide insights for the development of targeted preventive measures and the strategic allocation of resources, contributing to the reduction of the burden of arboviral diseases in Brazil.

## Data Availability

The data used in this research are available on the website https://datasus.saude.gov.br/transferencia-de-arquivos/.

## Supplementary Materials

The following supporting information can be downloaded at: https://www.mdpi.com/article/10.3390/v17020158/s1, Table S1: Week percentage change (WPC) of arbovirus infection in Brazil from 2023 and 2024.
