# Supplementary material for: Rising Incidence and Spatiotemporal Dynamics of Emerging and Reemerging Arboviruses in Brazil
Source: Viruses. 2025 Jan 24;17(2):158. doi: 10.3390/v17020158 (PMC11860164; doi:10.3390/v17020158)
Supplement: Supplementary file 1 [file viruses-17-00158-s001.zip › viruses-3403481-supplementary.pdf]

Table S1: Week percentage change (WPC) of arbovirus infection in Brazil from 2023 and 2024.

	Segment period	WPC	Lower CI	Upper CI	p-value
DENV	01-01-2023 to 03-26-2023	20.4207	14.3913	26.2334	**<0.000001**
DENV	03-26-2023 to 10-08-2023	-13.11982	-16.4366	-10.6793	**<0.000001**
DENV	10-08-2023 to 03-17-2024	26.4735	21.9989	31.1022	**<0.000001**
DENV	03-17-2024 to 09-01-2024	-15.57836	-18.4403	-12.8677	**<0.000001**
ZIKV	01-01-2023 to 03-12-2023	15.6004	10.4050	21.9236	**<0.000001**
ZIKV	03-12-2023 to 11-12-2023	-5.7304	-6.6063	-5.0134	**0.000040**
ZIKV	11-12-2023 to 03-17-2024	18.6632	15.7812	22.1910	**0.000040**
ZIKV	03-17-2024 to 08-11-2024	-11.3006	-12.6631	-9.6928	**0.000040**
ZIKV	08-11-2024 to 08-25-2024	-52.6055	-63.6403	-33.7337	**<0.000001**
CHIKV	01-01-2023 to 03-26-2023	11.1395	6.9998	16.2337	**<0.000001**
CHIKV	03-26-2023 to 10-08-2023	-9.7782	-11.1768	-8.6477	**<0.000001**
CHIKV	10-08-2023 to 03-03-2024	18.1754	15.9567	20.7039	**<0.000001**
CHIKV	03-17-2024 to 09-01-2024	-12.7916	-14.2436	-11.4415	**<0.000001**
OROV	01-01-2023 to 09-17-2023	-3.0371	-9.1219	3.4552	0.346238
OROV	09-17-2023 to 02-11-2024	34.2046	13.5020	58.6834	**0.000789**
OROV	02-11-2024 to 07-14-2024	-7.7695	-20.2465	6.6594	0.271243
OROV	07-14-2024 to 09-01-2024	-67.3537	-83.4462	-35.6170	**0.001554**

WPC =Week percent change. Dengue(DENV), Zika (ZIKV), and Chikungunya (CHIKV).
